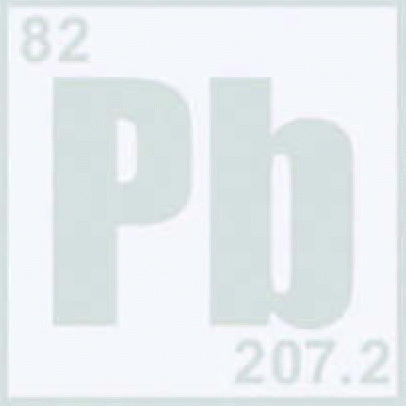# EHPnet: NIOSH Safety and Health Topic: Lead *and* Lead Exposure in Adults: A Guide for Health Care Providers

**Published:** 2007-01

**Authors:** Erin E. Dooley

Given the well-documented toxicity of lead, it is important to understand how to identify and avoid exposure. NIOSH and the New York State Department of Health are two entities that have posted lead-related information on the Internet in an effort to educate the public and clinicians about this health threat.

As part of its mission to educate the public about exposures to toxic materials in the workplace, NIOSH has pulled together a variety of resources on the topic of lead exposure and effects and made them available at **http://www.cdc.gov/niosh/topics/lead/**. This collection starts with a brief overview of how workers can be exposed to lead and the range of health effects caused by exposure. Next is a link to lead-related entries in the institute’s NIOSHTIC-2 bibliographic database of publications, grant reports, and journal articles supported in whole or part by NIOSH. Currently, there are more than 770 entries related to lead. The entries are arranged by date of publication, and most entries include links to the full text of the resource listed.

The NIOSH lead page also provides a link to the Adult Blood Lead Epidemiology Surveillance (ABLES) program page. This voluntary state-based program works to measure trends in adult blood lead levels and to minimize lead exposure. The ABLES program page lists the 37 participating state programs, with links to publications generated by each state. The page also has the latest compiled blood lead level data and a list of relevant publications, reports, and other resources.

The NIOSH lead page also lists selected publications as well as instructions from the *NIOSH Manual of Analytical Methods* for sampling and analysis of lead in different media. There are also numerous resources related to take-home lead exposure and its prevention. Lead poisoning, neurological effects, and mental retardation in family members have been linked to lead brought home by workers on their clothing and in vehicles.

A separate resource geared especially toward health care providers is available through the New York State Department of Health at **http://www.health.state.ny.us/nysdoh/lead/hlthcare.htm**. This page, which is also available in PDF form, provides a more in-depth overview of the adverse health effects of lead exposure. It briefly discusses the effects seen at different levels of lead exposure. It also provides information on the responsibilities of health care providers in reporting and evaluating elevated blood lead in patients they see, and advises clinicians on how to help their patients prevent dangerous exposures. The page outlines the New York State voluntary guidelines for controlling lead in workplaces and looks at how lead poisoning is treated clinically.

Clinicians can consult lists of exposure routes, including those associated with the workplace, hobbies (such as target shooting and stained-glass art), and use of substances (such as some folk remedies and moonshine whiskey). The page also has a rundown of six steps that health care providers can give to patients who work in places where lead is present to help reduce their own and their families’ exposure to lead.

## Figures and Tables

**Figure:**